# Impact of endometrial thickness and its combined effect with maternal age on singleton adverse neonatal outcomes in frozen–thawed embryo transfer cycles

**DOI:** 10.3389/fendo.2024.1430321

**Published:** 2025-01-14

**Authors:** Jie Wang, Lina Gao, Qiaoyun Huang, Weihua Jiang, Linjun Chen, Shanshan Wang

**Affiliations:** ^1^ Center for Reproductive Medicine and Obstetrics and Gynecology, Nanjing Drum Tower Hospital, Affiliated Hospital of Medical School, Nanjing University, Nanjing, China; ^2^ Center for Molecular Reproductive Medicine, Nanjing University, Nanjing, China

**Keywords:** frozen-thawed embryo transfer, singleton delivery, endometrial thickness, maternal age, adverse neonatal outcomes

## Abstract

**Background:**

Thin endometrial thickness (EMT) and advanced age are both common risk factors for adverse neonatal outcomes (ANOs). However, studies evaluating the impact of EMT and combined effect of EMT and age on ANOs remain scarce with conflicts.

**Method:**

A retrospective cohort study was conducted on 7,715 singleton deliveries from frozen embryo transfer (FET) cycles between 2017 and 2021. The participants were categorized into four groups based on EMT quartiles (≤8.5, 8.6–9.5, 9.6–10.5, and >10.5 mm). The primary outcome was preterm birth (PTB), and the secondary outcome included early PTB (EPTB), small/large for gestational age (SGA/LGA), and low birth weight (LBW).

**Results:**

Baseline characteristics were unevenly distributed across EMT groups. After adjusting for the confounders, EMT was found to be associated with the incidence of PTB (aOR 0.922, 95% CI 0.874–0.973, *p* = 0.003), EPTB (aOR 0.795, 95% CI 0.663–0.954, *p* = 0.014), LBW (aOR 0.886, 95% CI 0.796–0.986, *p* = 0.027), and LGA (aOR 1.038, 95% CI 1.004–1.074, *p* = 0.030). Furthermore, the rates of LBW in the group of EMT at 9.6–10.5 mm (aOR 0.551, 95% CI 0.339–0.895, *p* = 0.016) and >10.5 mm (aOR 0.536, 95% CI 0.332–0.865, *p* = 0.011) were lower compared to those with EMT ≤8.5 mm. Among women aged over 35, EMT of 9.6–10.5 mm was associated with a significantly lower incidence of LBW compared to thinner EMT, without increasing the risk of ANOs related to thicker EMT.

**Conclusions:**

Our study demonstrated the independent nonlinear impact of EMT on PTB, EPTB, LGA, and LBW. It provided new insights into the combined effects of EMT and age in FET cycles and offered valuable references for the clinical management and treatment strategies aimed at EMT.

## Introduction

The increasing utilization of frozen–thawed embryo transfer (FET) in clinical practice has significantly enhanced the cumulative pregnancy rate and reduced costs (1). In comparison to *in vitro* fertilization treatment/intracytoplasmic sperm injection (IVF/ICSI) cycles with fresh embryo transfer (ET), FET only requires the preparation of a receptive endometrium without the need to stimulate multiple follicular growth (2). Endometrial thickness (EMT) serves as a critical indicator of endometrial receptivity (3), representing a crucial factor in the advancement of assisted reproductive technology (ART) (4, 5). Previous studies predominantly focus on the impact of EMT in ART on pregnancy outcomes and perinatal complications, including implantation, live birth rate (LBR), miscarriage rate, ongoing pregnancy rate, and ectopic pregnancy (6).

LBR in ART has shown a remarkable increase over the past two decades, shifting the focus of studies away from solely achieving higher pregnancy rates (7). Attention has now turned toward adverse neonatal outcomes (ANOs) (8), which have been found to be significantly more common in pregnancies resulting from IVF compared to spontaneous pregnancies (9), such as preterm birth (PTB), low birth weight (LBW), small for gestational age (SGA), large for gestational age (LGA), and fetal macrosomia (FM) (10, 11).

However, there remains a scarcity of studies that address the association between EMT and ANOs in singleton pregnancies. For ET cycles, Du et al. identified EMT ≤7.5 mm on the human chorionic gonadotrophin (hCG) trigger day as an independent risk factor of LBW (12), while Wu et al. suggested an association between EMT and PTB and gestational age (GA), but not LBW (13). EMT was not found to be independently linked to adverse perinatal outcomes in intrauterine insemination cycles (14). In FET cycles, EMT below 8 mm, identified as a cutoff value, was associated with an increased risk of PTB and LBW (15). Additionally, advanced maternal age is a known high-risk factor for PTB and fetal growth restriction (FCR) (16) and is associated with both transfer outcomes and EMT (17).

Therefore, a retrospective cohort study was designed to investigate the correlations between EMT and ANOs in singleton deliveries resulting from FET cycles. The study aimed to assess the appropriate EMT to comprehensively optimize the neonatal outcomes across various age groups of women.

## Materials and methods

### Study population

**Figure 1:**
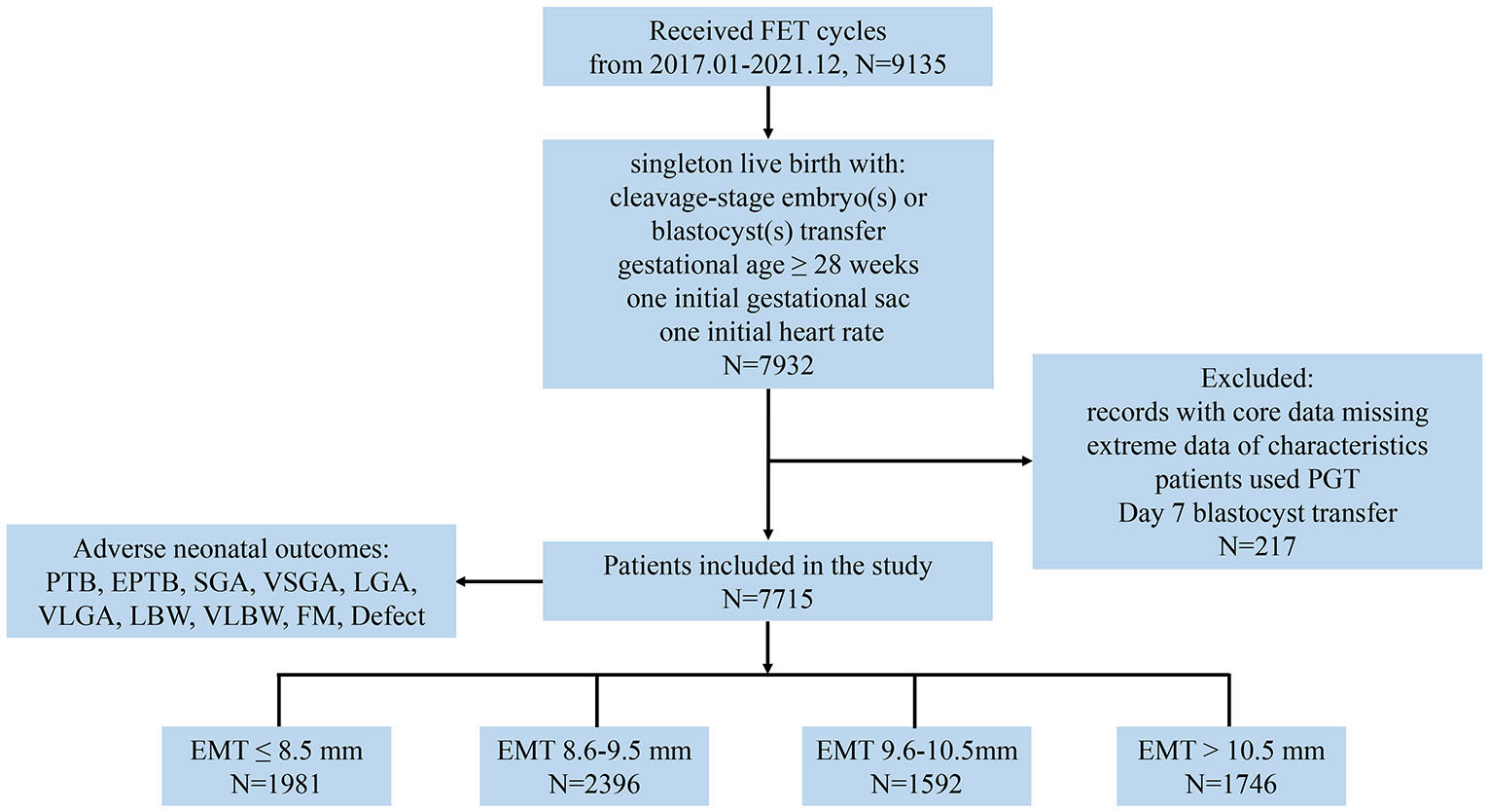
Study flow diagram. EMT, endometrial thickness; PTB, preterm birth; EPTB, early PTB; SGA, small for gestational age; VSGA, very SGA; LGA, large for gestational age; VLGA, very LGA; LBW, low birth weight; VLBW, very LBW; FM, fetal macrosomia.

This retrospective cohort study was conducted at the Center for Reproductive Medicine and Obstetrics and Gynecology, Nanjing Drum Tower Hospital, the Affiliated Hospital of Nanjing University Medical School in China, spanning from January 2017 to December 2021. The study included FET cycles where either one or two embryos were transferred, resulting in a single birth with the presence of one initial gestational sac and a heart rate at ≥28 weeks of GA for analysis. The exclusion criteria comprised records with missing core data, including baseline and outcome variables, cases with extreme values, patients who underwent preimplantation genetic testing (PGT), or those who had a day 7 blastocyst transfer. The patients were categorized into four groups based on the quartiles of EMT: ≤8.5, 8.6–9.5, 9.6–10.5, and >10.5 mm, respectively (**Figure 1**). This grouping approach not only mitigated random fluctuations and biases in the data but also enhanced the flexibility and robustness of regression models (18).

### Study procedures

The FET regimen was individualized according to the clinical characteristics and preferences of the patients. Different protocols were employed for endometrial preparation, such as nature cycle (NC), Gn stimulation cycle (Gn), mild stimulation cycle, or hormone replacement therapy (HRT) cycle.

In the NC protocol, transvaginal ultrasonography scans were utilized to monitor follicular development and endometrial thickness. Additionally, the serum levels of luteinizing hormone (LH), estradiol (E2), and progesterone (P) were monitored to determine the ovulation timing (19). In the Gn protocol, the patients received human menopausal gonadotropin (HMG) on day 2 of the menstrual cycle, with the dosage adjusted based on response after 4 to 5 days of stimulation (20). For the mild stimulation protocol, the patients were administered letrozole (2.5 mg orally once daily) or tamoxifen (20 mg orally once daily) for 5 days starting from the second or third day of menstruation, with HMG added based on follicular development assessed by serum sex hormones and ultrasound. Ovulation in the aforementioned protocols was induced with human chorionic gonadotropin (hCG) administration when the dominant follicle diameter exceeded 16 mm, along with dydrogesterone (Duphaston, Abbott, 20 mg twice daily for 4 or 6 days) to promote endometrial transformation. On the 5th and the 7th days after the hCG trigger, cleavage-stage embryos or blastocysts were thawed and transferred, respectively. Patients typically received Duphaston (20 mg dydrogesterone, b.i.d.) for luteal support, which was continued for 2 months following FET in pregnant patients.

In the HRT protocol, the patients had initiated treatment with oral estradiol (2 mg three times daily) for 14 days, commencing on the 2nd day of their menstrual cycle. Once the endometrial thickness reached the required standard (≥7 mm), oral estradiol combined with dydrogesterone compound tablets (2 mg estradiol and 10 mg dydrogesterone three times daily) was administered for 5 or 6 days, along with intramuscular progesterone (P) injections at 60 mg once daily for 5 or 6 days to induce endometrial transformation (21). Cleavage-stage embryos were thawed and transferred on the 5th day of endometrial transformation, while blastocysts were thawed and transferred on the 6th day. The patients generally received Femoston (2 mg estradiol and 10 mg dydrogesterone, t.i.d.) and P sustained-release vaginal gel (Crinone, Merck Serono, Switzerland, 90 mg, q.d.) for luteal support.

Embryo vitrification and warming procedures were performed according to the protocol of vitrification kits (KITAZATO) and warming kits (KITAZATO), respectively. The vitrificated embryos were preserved submerged in liquid nitrogen. At the day of transfer, the embryos were moved to thawing, washing solution, and culture media in proper order (22). Following a minimum culture period of 2 h, the embryos were transferred immediately after assessing embryonic survival using a microscope. Each patient underwent the transfer of a maximum of two embryos.

### Measurement of clinical features and outcomes

EMT was defined as the widest distance between the reflective endometrial–myometrial interfaces on transvaginal ultrasound, as evaluated by experienced sonographers (23). EMT was measured on the day of hCG trigger in NC, minimal stimulation and Gn stimulation cycle protocols, and from the final ultrasound before progesterone initiation in HRT cycles.

The baseline data of FET cycle characteristics were collected, including age and body mass index (BMI) of the couples, plasma levels of both basal follicle-stimulating hormone (FSH) and luteinizing hormone (LH), duration, type and cause infertility, number of embryos transferred, embryo stage, fertilization method, stimulation protocols, and pregnancy complications.

All live birth infants, who were delivered at ≥28 complete weeks of gestation, had recorded information of gender, weight, gestational age (GA), and birth defect. The primary outcome was preterm birth (PTB), and the secondary outcome included early PTB (EPTB), SGA, LGA, and LBW. The delivery that occurred before 32 and 37 weeks was defined as EPTB and PTB. Very LBW (VLBW), LBW, and FM were identified as live birth weight <1,500, <2,500, and >4,000 g at any GA, respectively. The GA of cleavage stage and blastocysts was calculated as days 17 and 19, respectively. The birth weight was standardized to account for the effect of GA and newborn gender by Z-score according to the Chinese birth weight reference at distinct GA (24). Z-score was calculated through the following equation: Z-score = (*x* - *μ*)/*σ*, in which *x* is the weight of an infant, *μ* is the average birthweight for the same gender and GA in the reference group, and *σ* is the standard deviation (SD) of the reference group. Very SGA (VSGA), SGA, LGA, and very LGA (VLGA) were identified as birth weight <3rd, <10th, >90^th^, and >97th percentiles, respectively. Additionally, birth defects were defined as congenital structural abnormalities, metabolic anomalies, deafness, tumor, or chromosomal defects.

### Statistical analysis

Clinical characteristics were recorded in the tables, with continuous data that were presented as median values and quartiles, while categorical indicators were summarized using absolute counts and relative frequencies. Unadjusted comparisons of continuous data in four groups of EMT were performed via the two-sided Wilcoxon rank-sum test. The differences of both exposure factors and outcome frequencies were calculated by two-sided *χ* ^2^ or Fisher’s exact test when appropriate.

Logistic regression model was employed to evaluate the correlations between the EMT and ANOs, including PTB, EPTB, SGA, VSGA, LGA, VLGA, LBW, VLBW, FM, and birth defect. To confirm whether EMT was an independent factor of the outcomes, potential confounders such as the baseline features for PTB, EPTB, SGA, VSGA, LGA, and VLGA and additional adjusted factors including PTB, EPTB, and GA for LBW, VLBW, and FM were added in the multivariable logistic regression analysis, which was used to calculate the adjusted odds ratio (aOR) with 95% confidence interval (CI). Smooth curve fitting graphed by EmpowerStats (www.empowerstats.com, X&Y Solutions, Inc., Boston, MA, USA) was performed to deal with non-linearity relationship between EMT and outcomes. Logistic regression analysis was also executed after propensity score matching (PSM) using nearest-neighbor method with a caliper of 0.03 and ratio of 1:1 to balance the baseline characteristics of the ANOs. Nomograms were developed based on multivariate logistic regression to evaluate the effect of EMT on predicting ANOs. *P* < 0.05 was considered significant for all hypothesis tests. All statistical analyses were managed by using software SPSS v22.0 and R v4.3.0.

## Results

### Baseline demographics and clinical features of patients in FET cycles

A total of 7,715 couples with singleton deliveries from FET cycles were included in the analysis. The live births were classified based on EMT as follows: EMT ≤8.5 mm (*N* = 1,981), 8.6 ≤ EMT ≤ 9.5 mm (*N* = 2,396), 9.6 ≤ EMT ≤ 10.5 mm (*N* = 1,592), and EMT >10.5 mm (*N* = 1,746), with the median (1st quartile, 3rd quartile) EMT value being 9.5 mm (8.5 mm, 10.5 mm).

The demographic particulars and principal cycle features are delineated in **Table 1**. With the exception of paternal age and BMI, as well as cycle rank and fertilization method, other characteristics were non-uniformly distributed across the four EMT groups. Women with thinner EMT tended to be older, exhibited lower BMI values, possessed elevated basal FSH levels and lower basal LH levels, endured shorter durations of infertility, had lower rates of HRT protocols, experienced higher incidences of secondary infertility, presented with more cases of tubal factor as the cause of infertility, and underwent double blastocyst transfers more frequently. In terms of neonatal outcomes, the participants were also categorized into four groups, with only LBW displaying a distinct distribution among the four EMT groups (**Supplementary Table S1**).

**Table 1:** Baseline characteristics according to EMT categorization.

Characteristics	Endometrial thickness				*p-*values
	≤8.5 mm	8.6–9.5 mm	9.6–10.5 mm	>10.5 mm	
	*N* = 1,981	*N* = 2,396	*N* = 1,592	*N* = 1,746	
Maternal age (years)	30 (28, 34)	30 (28, 33)	30 (28, 33)	30 (28, 34)	0.020^*^
Paternal age (years)	31 (29, 35)	31 (29, 35)	31 (29, 35)	31 (29, 35)	0.331
Maternal BMI	21.9 (20.1, 24.4)	22.2 (20.3, 24.6)	22.5 (20.4, 25.2)	22.9 (20.7, 25.3)	<0.001^*^
Paternal BMI	24.5 (22.2, 26.8)	24.3 (22.0, 26.9)	24.6 (22.2, 26.9)	24.5 (22.3, 27.0)	0.085
Basal FSH	7.18 (6.02, 8.62)	7.01 (5.86, 8.50)	6.96 (5.80, 8.32)	7.18 (5.98, 8.52)	0.001^*^
Basal LH	4.80 (3.53, 6.78)	5.14 (3.67, 7.18)	5.13 (3.60, 7.38)	5.03 (3.52, 7.35)	0.004^*^
Infertility duration (years)	3.0 (1.6, 4.0)	3.0 (2.0,4.0)	3.0 (2.0,4.0)	3.0 (2.0,4.0)	0.002^*^
Endometrial preparation					<0.001^*^
Gn stimulation	89 (4.5%)	34 (1.4%)	20 (1.3%)	19 (1.1%)	
Natural cycle	123 (6.2%)	151 (6.3%)	109 (6.8%)	193 (11.1%)	
Mild stimulation	119 (6.0%)	47 (2.0%)	28 (1.8%)	31 (1.7%)	
Hormone replacement therapy	1,650 (83.3%)	2,164 (90.3%)	1,435 (90.1%)	1,503 (86.1%)	
Cycle rank					0.181
1	829 (41.9%)	1059 (44.2%)	706 (44.3%)	797 (45.6%)	
2	811 (40.9%)	941 (39.3%)	651 (40.9%)	671 (38.5%)	
≥3	341 (17.2%)	396 (16.5%)	235 (14.8%)	278 (15.9%)	
Fertilization method					0.605
IVF	1,482 (74.8%)	1,770 (73.9%)	1,165 (73.2%)	1,313 (75.2%)	
ICSI	440 (22.2%)	568 (23.7%)	379 (23.8%)	389 (22.3%)	
IVF+ICSI	59 (3.0%)	58 (2.4%)	48 (3.0%)	44 (2.5%)	
Infertility type					<0.001^*^
Primary	868 (43.8%)	1,331 (55.6%)	956 (6.1%)	1,105 (63.3%)	
Secondary	1,114 (56.2%)	1,065 (44.4%)	636 (39.9%)	641 (36.7%)	
Infertility cause					<0.001^*^
Tubal factor	1,192 (60.2%)	1,325 (55.3%)	905 (56.8%)	946 (54.2%)	
PCOS	305 (15.4%)	441 (18.4%)	282 (17.7%)	311 (17.8%)	
Endometriosis	74 (3.7%)	115 (4.8%)	87 (5.5%)	142 (8.1%)	
Uterine factor	22 (1.1%)	26 (1.1%)	13 (0.8%)	19 (1.1%)	
Male factor	352 (17.8%)	427 (17.8%)	286 (18.0%)	293 (16.8%)	
Unknown	36 (1.8%)	62 (2.6%)	19 (1.2%)	35 (2.0%)	
Pregnancy complication					0.046^*^
No	1,613 (81.4%)	1,907 (79.6%)	1,261 (79.2%)	1,357 (77.7%)	
Yes	368 (18.6%)	489 (20.4%)	331 (20.8%)	389 (22.3%)	
Number of embryos transferred					0.009^*^
1	1,165 (58.8%)	1,470 (61.4%)	956 (60.1%)	1,118 (64.0%)	
2	816 (41.2%)	926 (38.6%)	636 (39.9%)	628 (36.0%)	
Embryo stage					<0.001^*^
Single blastocyst	974 (49.2%)	1206 (50.3%)	781 (49.1%)	911 (52.1%)	
Double blastocysts	348 (17.6%)	400 (16.7%)	220 (13.8%)	211 (12.1%)	
Single cleavage-stage embryo	191 (9.6%)	265 (11.1%)	175 (11.0%)	207 (11.9%)	
Double cleavage-stage embryos	468 (23.6%)	525 (21.9%)	416 (26.1%)	417 (23.9%)	

Data are presented as the median (quartiles) or number (percentage).

^*^ *p* value <0.05.

### Correlations between EMT and adverse neonatal outcomes

**Figure 2:**
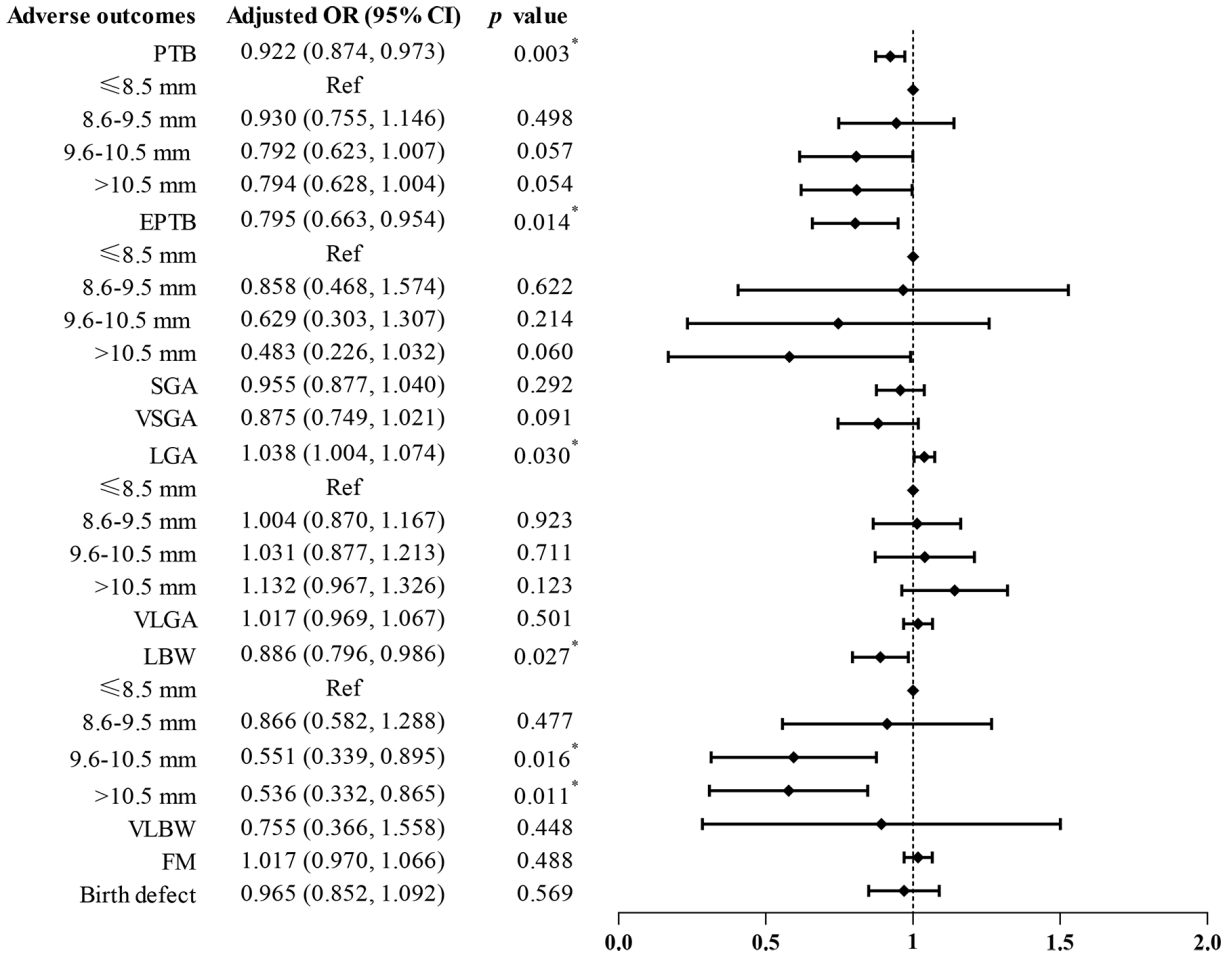
Adjusted odds ratios (95% confidence intervals) via multivariate logistic analysis for ANOs by EMT. Ref, reference.

To assess the influence of EMT on ANOs, we performed univariate and multivariate logistic regression analyses (**Supplementary Table S2** and **Figure 2**). The results after adjusting for confounding factors were basically consistent with those obtained from the univariate model. EMT was significantly negatively associated with an increasing incidence of PTB (aOR 0.922, 95% CI 0.874–0.973, *p* = 0.003), EPTB (aOR 0.795, 95% CI 0.663–0.954, *p* = 0.014), and LBW (aOR 0.886, 95% CI 0.796–0.986, *p* = 0.027) and was positively related to an increase in LGA rates (aOR 1.038, 95% CI 1.004–1.074, *p* = 0.030). Additionally, when EMT was regarded as a categorical variable, the PTB rates tended to be decreased among infants in the group of EMT of 9.6–10.5 mm (aOR 0.792, 95% CI 0.623–1.007) and >10.5 mm (aOR 0.794, 95% CI 0.628–1.004) compared to the group of patients with EMT ≤8.5 mm. Moreover, the incidence of LBW significantly decreased in patients with EMT of 9.6–10.5 mm (aOR 0.551, 95% CI 0.339–0.895, *p* = 0.016) and >10.5 mm (aOR 0.536, 95% CI 0.332–0.865, *p* = 0.011) than those of patients in the EMT ≤8.5 mm group. No significant differences were observed between EMT and the incidences of SGA, VSGA, VLGA, VLBW, FM, and birth defect. These findings suggested that EMT as a continuous variable was an effective factor of PTB, EPTB, LGA, and LBW, while EMT as a categorical indicator also independently influenced LBW.

**Figure 3:**
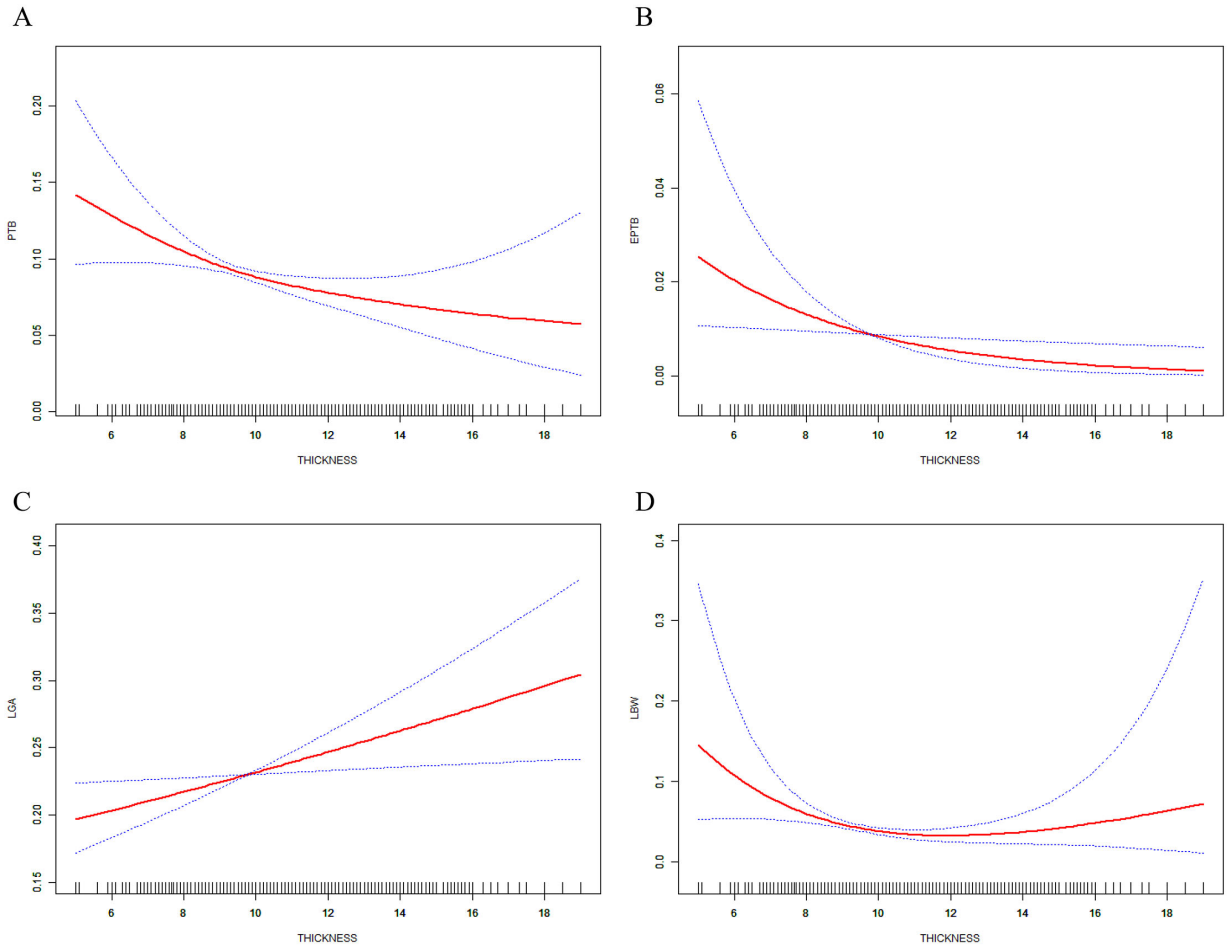
Relations between EMT and incidence of ANOs. **(A)** PTB, **(B)** EPTB, and **(C)** LGA, analysis was adjusted for maternal and paternal age and BMI, basal FSH and LH, infertility years, type and cause, cycle rank, fertilization method, pregnancy complication, number of embryos transferred, embryo stage, and endometrial preparation. **(D)** Adjusted was performed with additional factors including PTB, EPTB, and GA.

The predicted probabilities with 95% CI of PTB, EPTB, LGA, and LBW by EMT were revealed in smooth curve fitting (**Figure 3**). After adjusting for confounding factors, increased EMT was found to be associated with a decrease of PTB (**Figure 3A**) and EPTB (**Figure 3B**) and related to the increase of LGA (**Figure 3C**). The incidence of LBW, which was additionally adjusted with PTB, EPTB, and GA, exhibited a decrease with increasing EMT and then stayed steady when EMT ranged 8–14 mm and then tended to increase after EMT became excessively thicker (**Figure 3D**).

### Effect of EMT as a continuous variable on PTB, EPTB, LGA, and LBW

To investigate the factors affecting PTB, EPTB, LGA, and LBW, univariate logistic regression analysis was employed. Similar to EMT, infertility cause was also found to be associated with the aforementioned four outcomes, while pregnancy complication showed a tendency to be correlated with them. Maternal age and cycle rank were related to both PTB and LBW. Maternal BMI was associated with PTB, EPTB, and LGA. Interestingly, basal FSH revealed significant correlations with EPTB, LGA, and LBW (**Supplementary Table S3**).

In addition to multivariable logistic regression, PSM was also used to adjust for potential confounders. Following PSM, all baseline characteristics, except for EMT, were balanced between the populations with and without ANOs. The increase of EMT was still found to be significantly associated with a decreased risk of PTB (aOR 0.873, 95% CI 0.818–0.932, *p* < 0.001), EPTB (aOR 0.714, 95% CI 0.555–0.919, *p* = 0.009), and LBW (aOR 0.874, 95% CI 0.759–0.979, *p* = 0.006) as well as an increased risk of LGA (OR 1.044, 95% CI 1.002–1.087, *p* = 0.040) (**Table 2**). Overall, these results indicated that EMT was an independent factor influencing PTB, EPTB, LGA, and LBW.

**Table 2:** Associations between EMT and PTB, EPTB, LGA, and LBW after propensity score matching.

Adverse outcomes	Non-adjusted		PSM-adjusted	
	β (95% CI)	*p-*values	β (95% CI)	*p-*values
PTB	0.938 (0.893, 0.989)	0.016	0.873 (0.818, 0.932)	<0.001^*^
EPTB	0.823 (0.691, 0.981)	0.030	0.714 (0.555, 0.919)	0.009^*^
LGA	1.049 (1.015, 1.083)	0.004	1.044 (1.002, 1.087)	0.040^*^
LBW	0.864 (0.799, 0.933)	<0.001	0.874 (0.759, 0.979)	0.006^*^

^*^ *p* value <0.05.

The area under the curve (AUC) of PTB, EPTB, LGA, and LBW were 0.528, 0.569, 0.523, and 0.569, respectively, when using EMT as the sole indicator in the prediction of the abovementioned outcomes (**Supplementary Figures S1A–D**). Nomograms, DSA, and CIC were constructed accordingly to predict the risks of PTB, EPTB, LGA, and LBW using factors associated with each outcome in the univariate analysis (**Supplementary Table S3**). Women with thin EMT and pregnancy complication were more likely to experience PTB and had neonates with LBW (**Supplementary Figures S2A, D**). Overweight and obese increased the incidence of PTB and LGA (**Supplementary Figures S2A, C**). The impact of EMT in the prediction of EPTB and LGA appeared to be moderate (**Supplementary Figures S2B, C**). In conclusion, although EMT was an independent factor influencing ANOs such as PTB and LBW, relying solely on EMT was inadequate to construct a predictive model for ANOs. Nevertheless, EMT should be considered as a significant factor in these predictive models, due to its critical influence on ANOs.

### Combined effect of categorical EMT and maternal age on PTB and LBW

Thin EMT and advanced maternal age were both common findings in the cases of PTB and LBW (**Supplementary Table S3**), and maternal age was unevenly distributed across the four EMT groups (**Table 1**). Although maternal age was not the independent factor of ANOs in our cohort (**Supplementary Table S4**), the relationship between EMT and maternal age in terms of the incidence of PTB and LBW is presented in **Table 3** to analyze the combined effects.

**Table 3:** Correlations between EMT and maternal age in terms of the incidence of PTB and LBW.

	PTB (%)	Endometrial thickness				LBW (%)	Endometrial thickness			
		≤8.5 mm	8.6–9.5 mm	9.6–10.5 mm	>10.5 mm		≤8.5 mm	8.6–9.5 mm	9.6–10.5 mm	>10.5 mm
Maternal age	≤30	8.99%	8.33%	8.51%	7.80%	≤30	4.10%	4.08%	3.24%	2.94%
	31–35	10.15%	9.99%	8.21%	8.22%	31–35	6.82%	4.42%^a^	4.01%	3.67%
	36–38	11.64%	11.54%	5.79%	13.45%^b^	36–38	7.41%	7.69%	2.48%^c^	5.26%
	≥39	11.45%	14.52%	10.11%	8.47%	≥39	6.87%	7.26%	1.12%^c^	1.69%

a For women aged 31–35 years, changed EMT to more than 8.5 mm would be numerically beneficial in reducing LBW.

b The risk of PTB in women aged 36–38 years was increased when EMT was thicker than 10.5 mm.

c For women aged older than 35 years, EMT of 9.6–10.5 mm was appropriate to decrease LBW compared with thinner EMT but not to increase the incidence of PTB and LBW compared with thicker EMT.

There were no significant differences found in both PTB and LBW among EMT groups in patients aged 30 years or younger. For women aged 31–35 years with EMT of 8.6–9.5 mm, the LBW rate was significantly lower (4.42% vs. 6.82%, OR 0.633, 95% CI 0.402, 0.997, *p* = 0.047) compared to those with EMT ≤8.5 mm in the same age range. Regarding women aged 36–38 years, the LBW rate tended to decrease in EMT of 9.6–10.5 mm than EMT of 8.6–9.5 mm (2.48% vs.7.69%, OR 0.306, 95% CI 0.056, 1.102, *p* = 0.053), and the PTB rate significantly decreased with EMT of 9.6–10.5 mm than EMT of <10.5 mm (5.79% vs. 13.45%, OR 2.531, 95% CI 1.049, 6.105, *p* = 0.034). In terms of female patients aged 39 years or older, the rates of LBW were significantly lower with EMT of 9.6–10.5 mm than EMT of 8.6–9.5 mm (1.12% vs 7.26%, OR 0.146, 95% CI 0.003, 1.089, *p* = 0.048). In summary, the results suggested that EMT greater than 8.6 mm may be suitable for women aged 31–35 years to reduce LBW, and EMT of 9.6–10.5 mm could be an optimal option for female patients who have reached advanced age to decrease the incidence of LBW without increasing ANOs associated with overthickened EMT, such as PTB, LGA, and LBW itself.

## Discussion

The major objective of our study was to investigate the effect of EMT on ANOs in female patients undergoing FET cycles. The results indicated that reductions in EMT of female undergoing FET cycles adversely influenced the neonatal outcomes, leading to increased rates of PTB, EPTB, and LBW. Conversely, increases of EMT led to an increase of the incidence of LGA. No certain relationship was found between EMT and other outcomes. Previous studies have explored the association between EMT and ANOs, yielding conflicting findings. Chung et al. reported a 2.04-fold increased risk of LBW in EMT ≤10 mm compared with EMT >12 mm in ET cycles (25). However, Wu et al. found that EMT was the independent factor of PTB and GA but not LBW (13). In our study of FET cycles, EMT remained associated with LBW even after additional adjustments for GA and PTB. This association may be attributed to the significantly higher rates of LBW in ET cycles compared to FET cycles (26–28), possibly due to the superior endometrial receptivity in FET cycles (29, 30). Nevertheless, compared to the general population, the risks of LBW, SGA, and LGA remained poorer in FET cycles (31). In previous studies, lower birth weight was observed among women with EMT <9 mm (32), while PTB and LBW were found to increase when EMT <8 mm (15). In terms of LGA in FET cycles, a slight but significant increase in LGA was noted in the EMT 12–13.9 mm group compared with the thin group (33), and thin EMT was associated with higher rates of LBW and fewer cases of LGA (34). Overall, our study was the first to concurrently demonstrate that EMT was negatively correlated with both PTB and EPTB, independently influenced LBW even after rigorous adjustments, and positively associated with LGA.

The mechanism of EMT-influenced neonatal outcomes remains unclear. The placenta may be the most critical factor of perinatal outcomes and may also be influenced by the underlying endometrium (35). Decidualization of stromal cells and under-regulation of vascular endothelial growth factor were observed in female patients with thin EMT, leading to impaired placentation, a poorly vascularized endometrium, and insufficient oxygen supply for the embryo (36, 37). Abnormal angiogenesis of the endometrium in these patients can predispose them to PTB and abnormal placental development (38). LBW and PTB have been linked to decreased placental weight (PW) (39), as placental growth is anticipated to be proportional to birth weight (40). Blood pressure-mediated enhancement of the uterine–placental blood flow has been associated with gradual increases in birth weight, posing a risk factor for FCR and indicative of placental insufficiency (41). Changes in placental function may contribute to FCR or fetal overgrowth, leading to LGA infants (42, 43). The results and reasons of EMT associated with PTB, EPTB, LBW and LGA but not SGA in FET cycles needed to be further verified and explored in future studies.

To date, there are still no screening methods with high sensitivity that can accurately identify neonates at risk for PTB, EPTB, LGA, or LBW with high specificity to avoid unnecessary treatment and intervention costs. Consistent with previous studies, maternal BMI has been shown to be associated with PTB and LGA but not LBW (44), and low basal serum FSH levels have be related to a reduced risk of LBW in FET (45). In the present study, EMT was found to have a potential value in predicting adverse outcomes, particularly for PTB and LBW. In addition to cervical length, which is considered the most cost-effective method in clinical practice, uterocervical angle and placental strain have also been suggested to contribute to predicting PTB (46). A single third-trimester fetal and placental ultrasound combined with maternal characteristics has shown outstanding performance in assessing the risks of PTB, SGA, and LGA (47). Nevertheless, neither symphysis–fundal height nor ultrasonography has proven useful in predicting LBW (48). Incorporating additional features such as EMT, BMI, and basal FSH together into maternal characteristics may enhance the ability to identify ANOs, particularly in FET cycles of ART.

Interventions aimed at increasing endometrial thickness (EMT) are worth considering when dealing with a thin endometrium, as they may prove effective in enhancing pregnancy and neonatal outcomes (35). Additionally, many studies examining the relationship between EMT and outcomes in assisted reproductive technology (ART) did not take into account the potentially detrimental impact of age during endometrial recovery, which can be a time-consuming process in patients with a thin endometrium (49). Our findings suggest that EMT of 8.6 mm may be adequate for female patients aged 31–35 years, while the optimal EMT range of 9.6–10.5 mm is recommended for women over 35 years of age. EMT thicker than 9.6 mm may reduce the risks of PTB and LBW, whereas EMT thicker than 10.5 mm may lead to an increased incidence of PTB, LGA, and LBW (**Table 3**, **Figure 3**). For women approaching advanced age (35 years old), it is advisable to consider preparing a thicker EMT in advance. Furthermore, it is essential to consider the effects of both thin and thick EMT on ANOs.

This retrospective study has several limitations. Firstly, the findings require validation in independent cohorts. Secondly, there may be potential selection bias and unknown confounding factors that need further adjustment. While adjustments have been made for available confounding factors, certain variables such as basal levels of estrogen and anti-Müllerian hormone, adjuvant treatments for thin endometrium, maternal lifestyle habits, and nutritional status have not been accounted for. Thirdly, the sample sizes for VSGA, VLBW, and EPTB are small, which hinders a comprehensive elucidation of their characteristics.

In conclusion, the present retrospective study has demonstrated that EMT independently affects PTB, EPTB, LGA, and LBW in singleton deliveries resulting from FET cycles. Our study provides recommendations regarding appropriate EMT levels for older women undergoing assisted reproduction techniques to effectively reduce adverse neonatal outcomes. The impact and clinical significance of EMT in ANOs necessitate further validation and exploration through large-scale prospective studies.

## Data Availability

The raw data supporting the conclusions of this article will be made available by the authors, without undue reservation.

## Supplementary material

Supplementary Figure 1 Receiver operating characteristic curve of EMT as the only indicator for ANOs. **(A)** PTB, **(B)** EPTB, **(C)** LGA, and **(D)** LBW, respectively.

Supplementary Figure 2 Logistic regression nomogram to predict ANOs in FET. Factors of four nomograms were chosen based on the significant elements of univariate logistic regression in **(A)** PTB, **(B)** EPTB, **(C)** LGA, and (**D**) LBW, respectively.
