# Supplementary figures and images for: Impact of endometrial thickness and its combined effect with maternal age on singleton adverse neonatal outcomes in frozen–thawed embryo transfer cycles
Source: Front Endocrinol (Lausanne). 2025 Jan 14;15:1430321. doi: 10.3389/fendo.2024.1430321 (PMC11772174; doi:10.3389/fendo.2024.1430321)

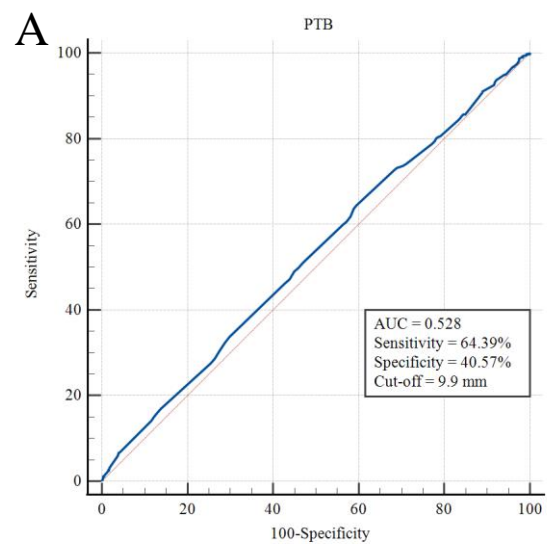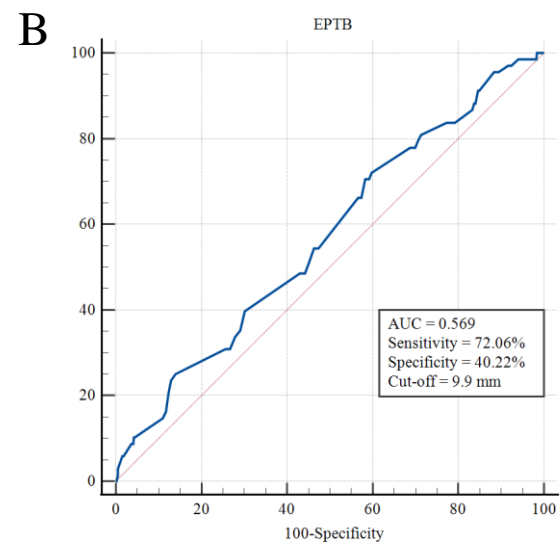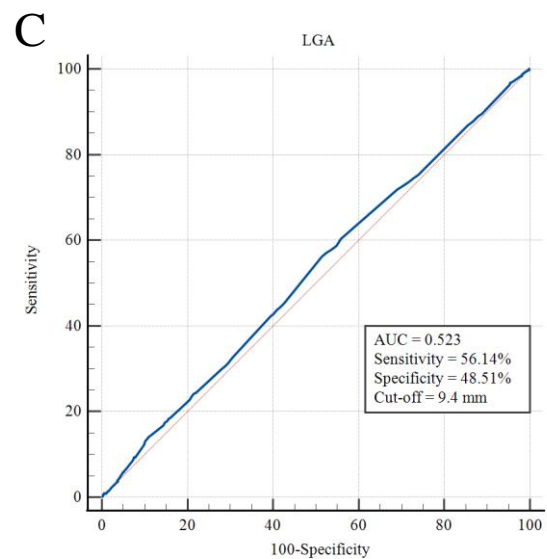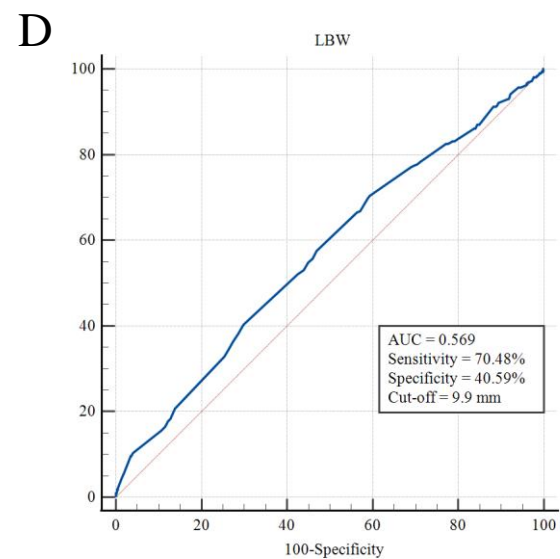

Supplement: Supplementary Figure 1 — Receiver operating characteristic curve of EMT as the only indicator for ANOs. (A) PTB, (B) EPTB, (C) LGA, and (D) LBW, respectively. [file DataSheet1.pdf]

A

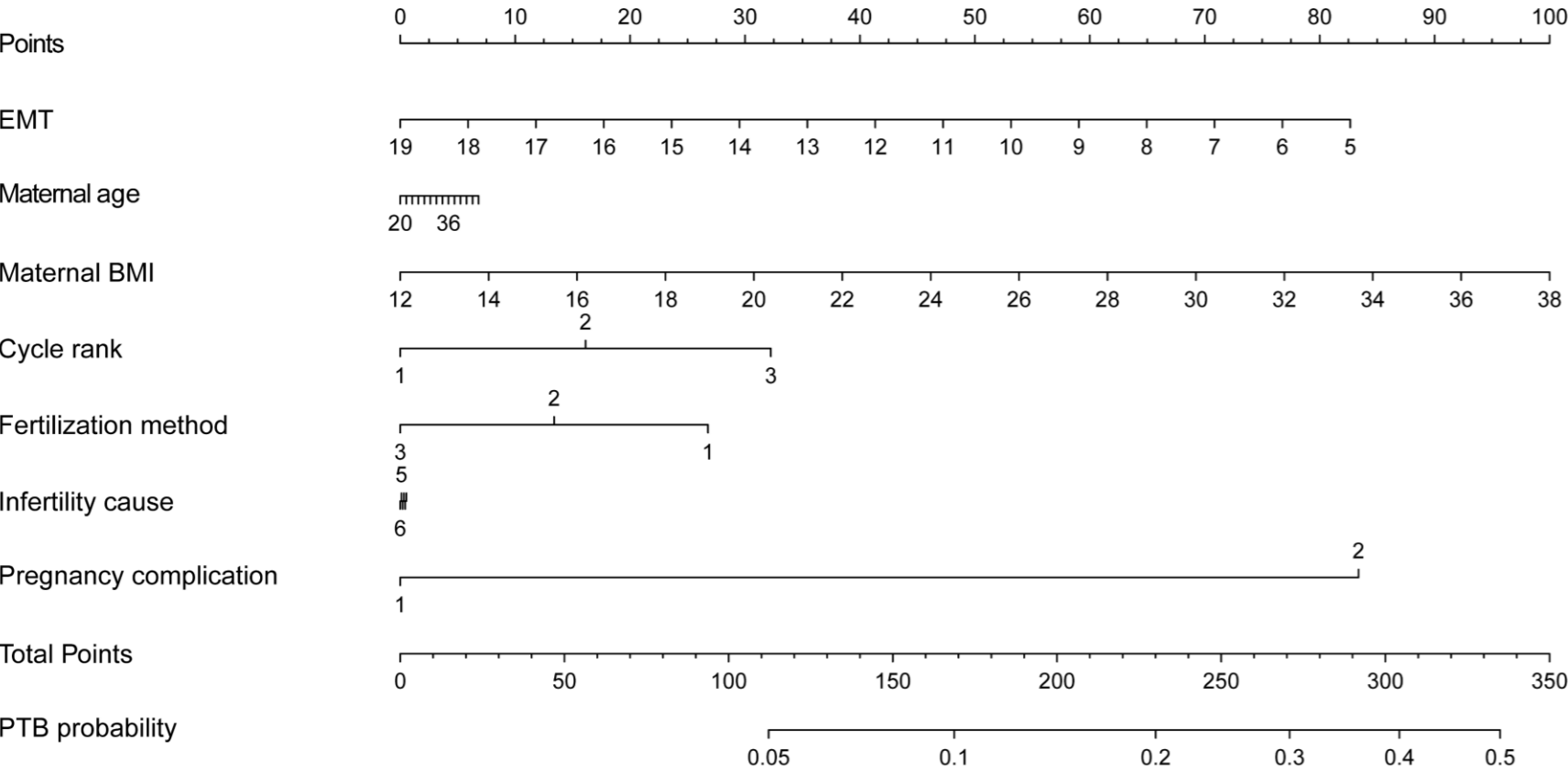

B

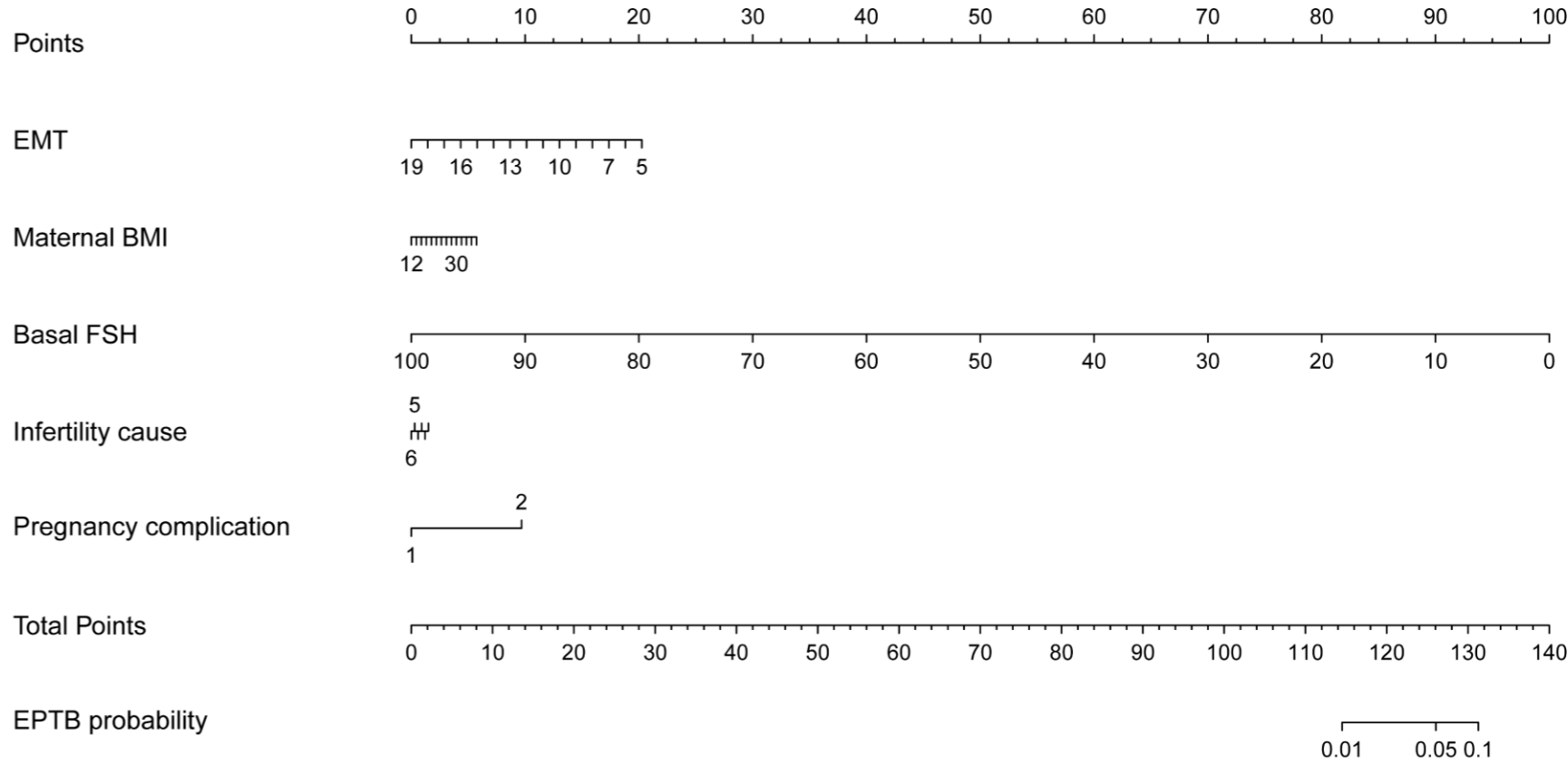

C

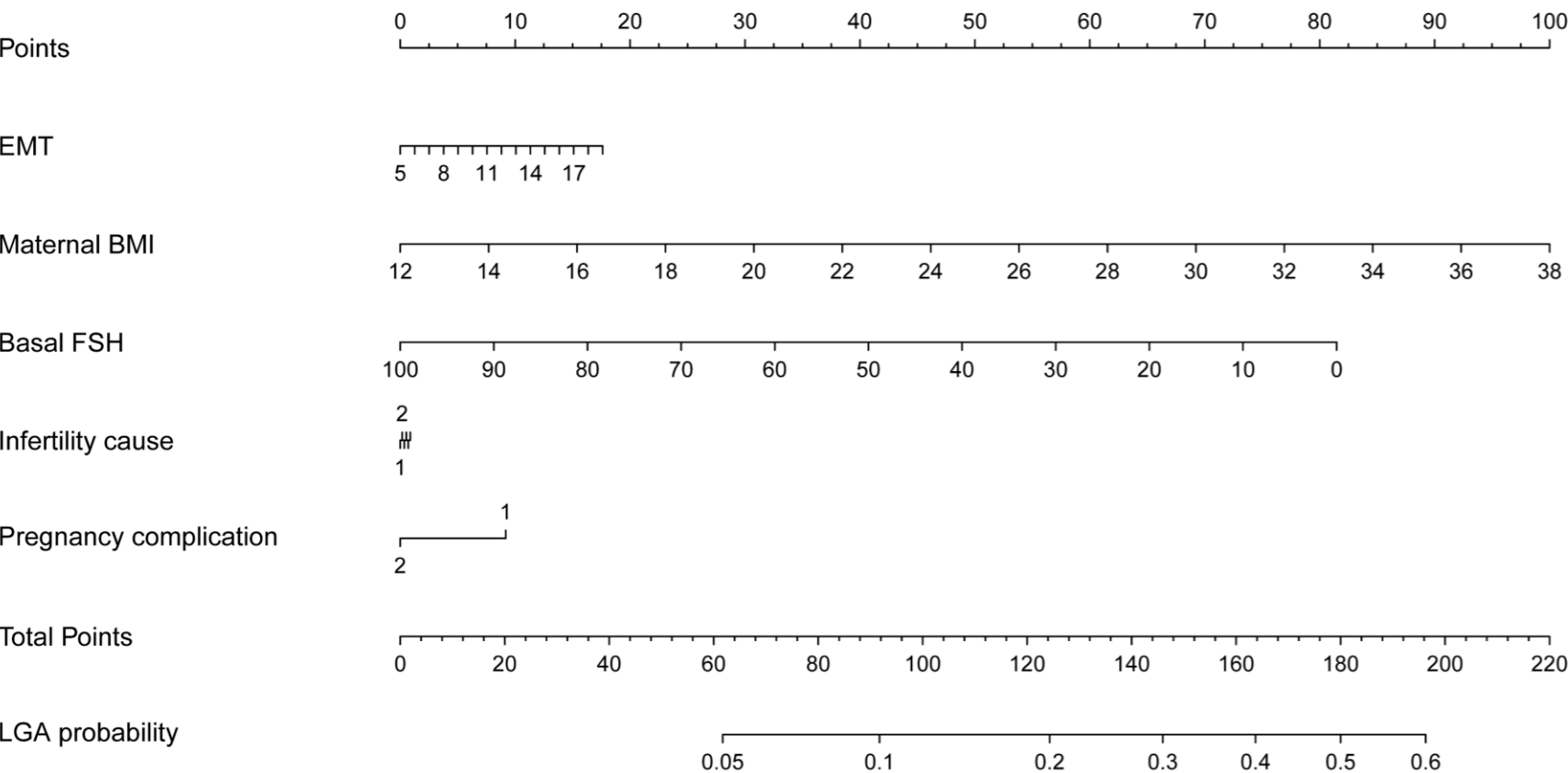

D

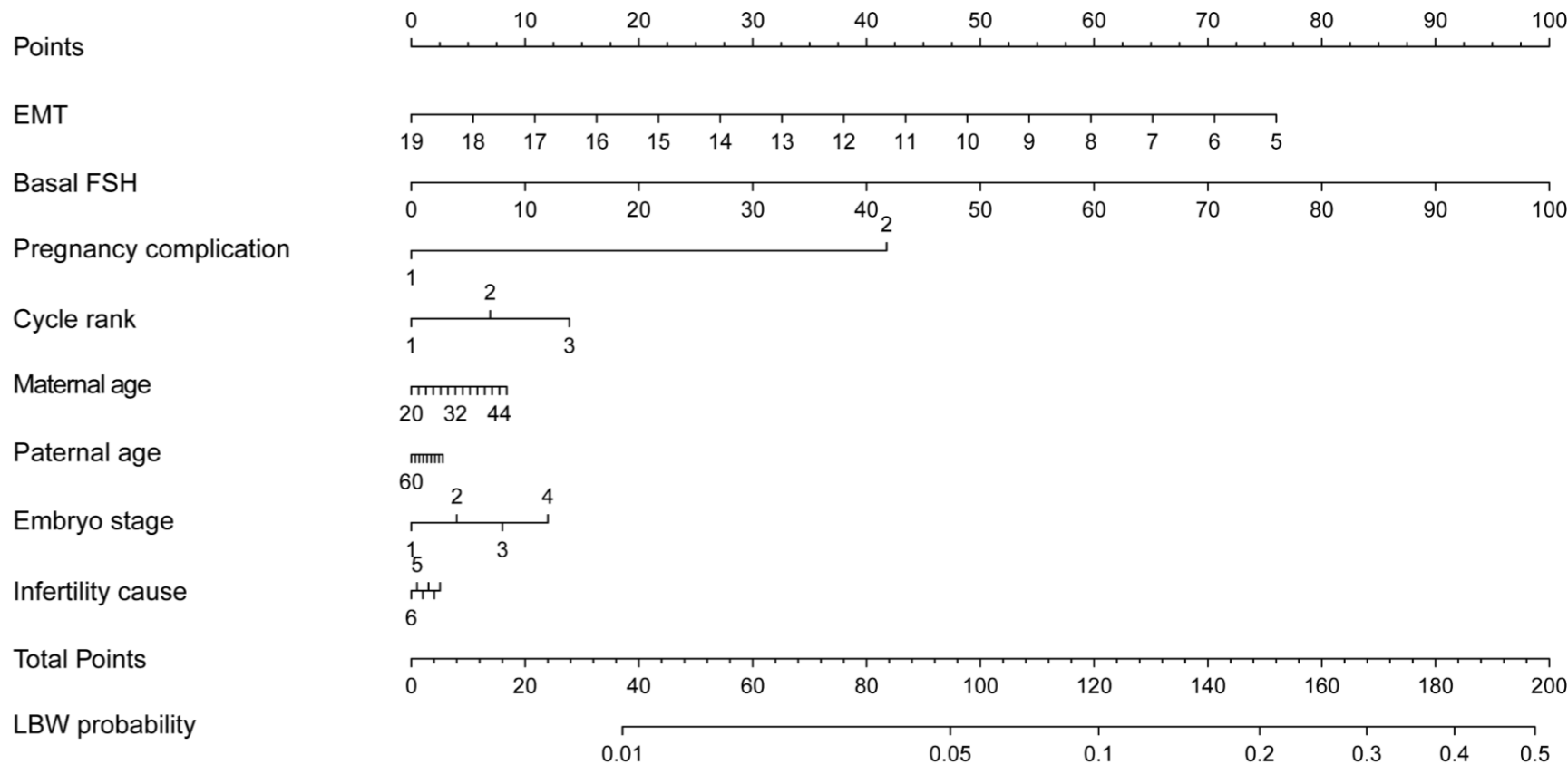

Supplement: Supplementary Figure 2 — Logistic regression nomogram to predict ANOs in FET. Factors of four nomograms were chosen based on the significant elements of univariate logistic regression in (A) PTB, (B) EPTB, (C) LGA, and (D) LBW, respectively. [file DataSheet2.pdf]
