# Supplementary material for: Impact of endometrial thickness and its combined effect with maternal age on singleton adverse neonatal outcomes in frozen–thawed embryo transfer cycles
Source: Front Endocrinol (Lausanne). 2025 Jan 14;15:1430321. doi: 10.3389/fendo.2024.1430321 (PMC11772174; doi:10.3389/fendo.2024.1430321)
Supplement: Supplementary file 3 [file Table1.docx]

Supplementary Table S1 Adverse neonatal outcomes by EMT category

| Outcomes | Endometrial thickness | | | | *p-*values |
| --- | --- | --- | --- | --- | --- |
|  | ≤8.5 mm | 8.6-9.5 mm | 9.6-10.5 mm | >10.5 mm |  |
|  | N=1981 | N=2396 | N=1592 | N=1746 |  |
| Gender |  |  |  |  | 0.523 |
| Male | 1090 (55.0%) | 1342 (56.0%) | 900 (56.5%) | 947 (54.2%) |  |
| Female | 891 (45.0%) | 1054 (44.0%) | 692 (43.5%) | 799 (45.8%) |  |
| BW (kg) | 3.4 (3.1, 3.7) | 3.4 (3.1, 3.7) | 3.4 (3.1, 3.7) | 3.4 (3.1, 3.7) | 0.585 |
| Z-score | 0.54 (-0.12, 1.20) | 0.55 (-0.19, 1.21) | 0.55 (-0.10, 1.21) | 0.55 (-0.09, 1.29) | 0.610 |
| VLBW (<1500g) | 11 (0.6%) | 10 (0.4%) | 5 (0.3%) | 3 (0.2%) | 0.263 |
| LBW (<2500g) | 109 (5.5%) | 112 (4.7%) | 53 (3.3%) | 58 (3.3%) | 0.001^*^ |
| FM (>4000g) | 220 (11.1%) | 254 (10.6%) | 167 (10.5%) | 199 (11.4%) | 0.795 |
| GA (week) | 39 (38, 39) | 39 (38, 39) | 39 (38, 39) | 39 (38, 39) | 0.318 |
| EPTB (<32 weeks) | 21 (1.1%) | 24 (1.0%) | 12 (0.8%) | 11 (0.6%) | 0.444 |
| PTB (<37 weeks) | 194 (9.8%) | 227 (9.5%) | 132 (8.3%) | 149 (8.5%) | 0.322 |
| VSGA (<3rd percentile) | 29 (1.5%) | 29 (1.2%) | 13 (0.8%) | 15 (0.9%) | 0.194 |
| SGA (<10th percentile) | 75 (3.8%) | 80 (3.3%) | 45 (2.8%) | 49 (2.8%) | 0.274 |
| LGA (>90th percentile) | 436 (22.0%) | 534 (22.3%) | 369 (23.2%) | 435 (24.9%) | 0.143 |
| VLGA (>97th percentile) | 195 (9.8%) | 227 (9.5%) | 157 (9.9%) | 178 (10.2%) | 0.895 |
| Defect |  |  |  |  | 0.777 |
| Yes | 32 (1.6%) | 36 (1.5%) | 20 (1.3%) | 29 (1.7%) |  |
| No | 1949 (98.4%) | 2360 (98.5%) | 1572 (98.7%) | 1717 (98.3%) |  |

*Note*: Data are presented as the number (percentage) or median (quartiles).

^*^: Indicates a *p* value <0.05.
