# Supplementary material for: Impact of endometrial thickness and its combined effect with maternal age on singleton adverse neonatal outcomes in frozen–thawed embryo transfer cycles
Source: Front Endocrinol (Lausanne). 2025 Jan 14;15:1430321. doi: 10.3389/fendo.2024.1430321 (PMC11772174; doi:10.3389/fendo.2024.1430321)
Supplement: Supplementary file 4 [file Table2.docx]

Supplementary Table S2 Univariate logistic analysis for ANOs by EMT

| Adverse neonatal outcomes | Non-adjusted β/OR | *p-*values |
| --- | --- | --- |
|  | (95% CI) |  |
| PTB (<37 weeks) | 0.938 (0.893, 0.989) | 0.016 |
| PTB (categorized into 4 groups) |  | 0.323 |
| ≤8.5 mm (N=1981) | Ref |  |
| 8.6-9.5 mm (N=2396) | 0.964 (0.788, 1.179) | 0.722 |
| 9.6-10.5 mm (N=1592) | 0.833 (0.611, 1.050) | 0.122 |
| >10.5 mm (N=1746) | 0.859 (0.687, 1.075) | 0.185 |
| EPTB (<32 weeks) | 0.823 (0.691, 0.981) | 0.030 |
| EPTB (categorized into 4 groups) |  | 0.451 |
| ≤8.5 mm (N=1981) | Ref |  |
| 8.6-9.5 mm (N=2396) | 0.944 (0.524, 1.701) | 0.849 |
| 9.6-10.5 mm (N=1592) | 0.709 (0.348, 1.445) | 0.344 |
| >10.5 mm (N=1746) | 0.592 (0.284, 1.231) | 0.160 |
| SGA (<10th percentile) | 0.930 (0.855, 1.012) | 0.093 |
| VSGA (<3rd percentile) | 0.856 (0.736, 0.996) | 0.044 |
| LGA (>90th percentile) | 1.049 (1.015, 1.083) | 0.004 |
| LGA (categorized into 4 groups) |  | 0.143 |
| ≤8.5 mm (N=1981) | Ref |  |
| 8.6-9.5 mm (N=2396) | 1.016 (0.881, 1.173) | 0.826 |
| 9.6-10.5 mm (N=1592) | 1.069 (0.913, 1.252) | 0.406 |
| >10.5 mm (N=1746) | 1.176 (1.010, 1.369) | 0.037 |
| VLGA (>97th percentile) | 1.030 (0.984, 1.079) | 0.204 |
| LBW (<2500g) | 0.864 (0.799, 0.933) | <0.001 |
| LBW (categorized into 4 groups) |  | 0.002 |
| ≤8.5 mm (N=1981) | Ref |  |
| 8.6-9.5 mm (N=2396) | 0.842 (0.642, 1.104) | 0.214 |
| 9.6-10.5 mm (N=1592) | 0.591 (0.423, 0.827) | 0.002 |
| >10.5 mm (N=1746) | 0.590 (0.426, 0.817) | 0.001 |
| VLBW (<1500g) | 0.698 (0.518, 0.940) | 0.018 |
| FM (>4000g) | 1.030 (0.986, 1.076) | 0.187 |
| Birth defect | 0.953 (0.846, 1.074) | 0.428 |
