# Supplementary material for: Impact of endometrial thickness and its combined effect with maternal age on singleton adverse neonatal outcomes in frozen–thawed embryo transfer cycles
Source: Front Endocrinol (Lausanne). 2025 Jan 14;15:1430321. doi: 10.3389/fendo.2024.1430321 (PMC11772174; doi:10.3389/fendo.2024.1430321)
Supplement: Supplementary file 5 [file Table3.docx]

Supplementary Table S3 Univariate logistic regression of impact factors on PTB, EPTB, LGA and LBW

| Factors | Values | PTB | | EPTB | | LGA | | LBW | |
| --- | --- | --- | --- | --- | --- | --- | --- | --- | --- |
|  | median (quartiles) / n (%) | β (95% CI) | *p-* values | β (95% CI) | *p-* values | β (95% CI) | *p-* values | β (95% CI) | *p-* values |
| Maternal age (years) | 30 (28, 33) | 1.025 (1.007, 1.044) | 0.006^*^ | 1.046 (0.991, 1.104) | 0.100 | 1.000 (0.987, 1.012) | 0.964 | 1.037 (1.012, 1.064) | 0.004^*^ |
| Paternal age (years) | 31 (29, 35) | 1.012 (0.997, 1.027) | 0.124 | 1.007 (0.962, 1.055) | 0.762 | 1.002 (0.991, 1.012) | 0.755 | 1.023 (1.002, 1.044) | 0.031^*^ |
| Maternal BMI | 22.3 (20.3, 24.8) | 1.069 (1.042, 1.093) | <0.001^*^ | 1.071 (1.001, 1.147) | 0.048^*^ | 1.101 (1.083, 1.118) | <0.001^*^ | 1.018 (0.985, 1.052) | 0.283 |
| Paternal BMI | 24.5 (22.1, 26.9) | 1.007 (0.986, 1.029) | 0.513 | 0.936 (0.872, 1.004) | 0.063 | 1.042 (1.027, 1.057) | <0.001^*^ | 0.995 (0.965, 1.026) | 0.739 |
| Basal FSH | 7.08 (5.90, 8.50) | 0.995 (0.969, 1.021) | 0.688 | 0.848 (0.753, 0.955) | 0.007^*^ | 0.965 (0.946, 0.984) | <0.001^*^ | 1.030 (1.004, 1.057) | 0.022^*^ |
| Basal LH | 5.01 (3.60, 7.17) | 1.005 (0.989, 1.021) | 0.563 | 1.019 (0.978, 1.061) | 0.372 | 0.994 (0.982, 1.006) | 0.303 | 1.007 (0.985, 1.029) | 0.562 |
| Infertility duration (years) | 3.0 (2.0, 4.0) | 1.032 (1.000, 1.064) | 0.050 | 0.927 (0.826, 1.040) | 0.198 | 1.007 (0.985, 1.029) | 0.551 | 0.988 (0.942, 1.036) | 0.617 |
| Stimulation protocols |  |  | 0.639 |  | 0.853 |  | 0.120 |  | 0.407 |
| Hormone replacement therapy | 6752 (87.5%) | Ref |  | Ref |  | Ref |  | Ref |  |
| Gonadotropin | 162 (2.1%) | 1.082 (0.642, 1.825) | 0.768 | 1.443 (0.349, 5.958) | 0.613 | 0.804 (0.544, 1.189) | 0.275 | 1.482 (0.733, 2.841) | 0.236 |
| Natural cycle | 576 (7.5%) | 0.817 (0.594, 1.123) | 0.212 | 1.011 (0.404, 2.530) | 0.982 | 0.806 (0.652, 0.997) | 0.047^*^ | 0.895 (0.575, 1.392) | 0.621 |
| Minimal stimulation | 225 (2.9%) | 1.016 (0.644, 1.605) | 0.945 | 1.560 (0.485, 5.016) | 0.456 | 0.840 (0.604, 1.168) | 0.298 | 1.381 (0.779, 2.448) | 0.269 |
| Cycle rank |  |  | <0.001^*^ |  | 0.424 |  | 0.315 |  | <0.001^*^ |
| 1 | 3391 (44.0%) | Ref |  | Ref |  | Ref |  | Ref |  |
| 2 | 3074 (39.8%) | 1.334 (1.121, 1.588) | 0.001^*^ | 0.985 (0.573, 1.693) | 0.956 | 1.049 (0.935, 1.178) | 0.414 | 1.192 (0.928, 1.532) | 0.169 |
| ≥3 | 1250 (16.2%) | 1.594 (1.286, 1.978) | <0.001^*^ | 1.459 (0.777, 2.740) | 0.240 | 0.930 (0.795, 1.088) | 0.364 | 1.744 (1.302, 2.337) | <0.001^*^ |
| Fertilization method |  |  | 0.024^*^ |  | 0.138 |  | 0.755 |  | 0.379 |
| IVF | 5730 (74.3%) | Ref |  | Ref |  | Ref |  | Ref |  |
| ICSI | 1776 (23.0%) | 0.761 (0.625, 0.927) | 0.007^*^ | 0.490 (0.242, 0.989) | 0.047^*^ | 1.049 (0.925, 1.190) | 0.454 | 0.822 (0.624, 1.083) | 0.164 |
| IVF+ICSI | 209 (2.7%) | 0.886 (0.542, 1.448) | 0.628 | 0 (0, NA) | 0.995 | 1.010 (0.727, 1.402) | 0.953 | 0.958 (0.486, 1.891) | 0.902 |
| Infertility type |  |  |  |  |  |  |  |  |  |
| Primary | 4259 (55.2%) | Ref |  | Ref |  | Ref |  | Ref |  |
| Secondary | 3456 (44.8%) | 1.124 (0.962, 1.313) | 0.140 | 1.028 (0.636, 1.663) | 0.910 | 1.081 (0.972, 1.202) | 0.152 | 0.935 (0.750, 1.166) | 0.552 |
| Infertility cause |  |  | <0.001^*^ |  | 0.005^*^ |  | 0.029^*^ |  | <0.001^*^ |
| Tubal factor | 4368 (56.6%) | Ref |  | Ref |  | Ref |  | Ref |  |
| PCOS | 1339 (17.4%) | 1.244 (1.018, 1.520) | 0.033^*^ | 1.547 (0.866, 2.764) | 0.140 | 1.177 (1.021, 1.356) | 0.024^*^ | 1.049 (0.779, 1.412) | 0.753 |
| Endometriosis | 418 (5.4%) | 0.922 (0.642, 1.323) | 0.658 | 1.163 (0.412, 3.282) | 0.776 | 0.821 (0.638, 1.057) | 0.127 | 1.065 (0.657, 1.726) | 0.799 |
| Uterine factor | 80 (1.0%) | 3.142 (1.858, 5.312) | <0.001^*^ | 6.333 (2.200, 18.236) | 0.001^*^ | 0.545 (0.287, 1.033) | 0.063 | 4.338 (2.353, 7.998) | <0.001^*^ |
| Male factor | 1358 (17.6%) | 0.784 (0.623, 0.987) | 0.039^*^ | 0.534 (0.225, 1.270) | 0.156 | 1.023 (0.885,1.183) | 0.755 | 0.819 (0.593, 1.132) | 0.227 |
| Unknown | 152 (2.0%) | 0.635 (0.321, 1.255) | 0.191 | 0.797 (0.109, 5.851) | 0.823 | 1.099 (0.754, 1.603) | 0.623 | 0.760 (0.308, 1.877) | 0.552 |
| Pregnancy complication |  |  |  |  |  |  |  |  |  |
| No | 6138 (79.6%) | Ref |  | Ref |  | Ref |  | Ref |  |
| Yes | 1577 (20.4%) | 3.196 (2.719, 3.758) | <0.001^*^ | 4.199 (2.601, 6.778) | <0.001^*^ | 0.889 (0.777, 1.017) | 0.086 | 3.265 (2.609, 4.086) | <0.001^*^ |
| Number of embryos transferred |  |  |  |  |  |  |  |  |  |
| 1 | 4709 (61.0%) | Ref |  | Ref |  | Ref |  | Ref |  |
| 2 | 3006 (39.0%) | 0.896 (0.763, 1.052) | 0.180 | 1.097 (0.676, 1.7833) | 0.707 | 1.012 (0.908, 1.128) | 0.833 | 1.179 (0.944, 1.473) | 0.146 |
| Embryo stage |  |  | 0.205 |  | 0.546 |  | 0.205 |  | 0.001^*^ |
| Single blastocyst | 3872 (50.2%) | Ref |  | Ref |  | Ref |  | Ref |  |
| Double blastocyst | 1179 (15.3%) | 1.020 (0.816, 1.276) | 0.861 | 0.985 (0.466, 2.081) | 0.969 | 0.998 (0.856, 1.164) | 0.981 | 1.396 (1.017, 1.915) | 0.039^*^ |
| Single cleavage | 838 (10.9%) | 1.137 (0.888, 1.456) | 0.308 | 1.547 (0.753, 3.176) | 0.235 | 0.821 (0.683, 0.987) | 0.036^*^ | 1.892 (1.368, 2.617) | <0.001^*^ |
| Double cleavage | 1826 (23.7%) | 0.853 (0.698, 1.043) | 0.121 | 1.347 (0.756, 2.399) | 0.312 | 0.962 (0.843, 1.098) | 0.565 | 1.341 (1.017, 1.769) | 0.038^*^ |
| EMT (mm) | 9.5 (8.5, 10.5) | 0.938 (0.893, 0.989) | 0.016^*^ | 0.823 (0.691, 0.981) | 0.030^*^ | 1.049 (1.015, 1.083) | 0.004^*^ | 0.864 (0.799, 0.933) | <0.001^*^ |
| EMT (categorized into 4 groups) |  |  | 0.323 |  | 0.451 |  | 0.143 |  | 0.002^*^ |
| ≤8.5 mm | 2981 (25.7%) | Ref |  | Ref |  | Ref |  | Ref |  |
| 8.6-9.5 mm | 2396 (31.1%) | 0.964 (0.788, 1.179) | 0.722 | 0.944 (0.524, 1.701) | 0.849 | 1.016 (0.881, 1.173) | 0.826 | 0.842 (0.642, 1.104) | 0.214 |
| 9.6-10.5 mm | 1592 (20.6%) | 0.833 (0.611, 1.050) | 0.122 | 0.709 (0.348, 1.445) | 0.344 | 1.069 (0.913, 1.252) | 0.406 | 0.591 (0.423, 0.827) | 0.002^*^ |
| >10.5 mm | 1746 (22.6%) | 0.859 (0.687, 1.075) | 0.185 | 0.592 (0.284, 1.231) | 0.160 | 1.176 (1.010, 1.369) | 0.037 | 0.590 (0.426, 0.817) | 0.001^*^ |

^*^: Indicates a *p* value <0.05.
