# Supplementary material for: Impact of endometrial thickness and its combined effect with maternal age on singleton adverse neonatal outcomes in frozen–thawed embryo transfer cycles
Source: Front Endocrinol (Lausanne). 2025 Jan 14;15:1430321. doi: 10.3389/fendo.2024.1430321 (PMC11772174; doi:10.3389/fendo.2024.1430321)
Supplement: Supplementary file 6 [file Table4.docx]

Supplementary Table S4 Multivariate logistic analysis for ANOs by maternal age

| Adverse neonatal outcomes | Adjusted β/OR | *p-*values |
| --- | --- | --- |
|  | (95% CI) |  |
| PTB | 1.018 (0.988, 1.048) | 0.248 |
| EPTB | 1.013 (0.975, 1.102) | 0.331 |
| LGA | 0.998 (0.978, 1.019) | 0.875 |
| LBW | 0.994 (0.939, 1.053) | 0.849 |
